# Tuning the Fe‐Oxide Nanoparticle Properties by Playing with Salt Precursors and *Camellia sinensis* Extract Concentrations

**DOI:** 10.1002/cphc.202500226

**Published:** 2025-07-23

**Authors:** Renzo Rueda‐Vellasmin, Juan A. Ramos‐Guivar, Jeferson Marques Santos, Noemi‐Raquel Checca‐Huaman, Edson C. Passamani

**Affiliations:** ^1^ Programa de Pós‐graduação em Física (PPGFis), Universidade Federal do Espírito Santo (Ufes) Programa de Pós‐graduação em Física da Universidade Federal do Espírito Santo Av. Fernando Ferrari, 514 Vitoria 29075‐910 ES Brazil; ^2^ Grupo de Investigación de Nanotecnología Aplicada para Biorremediación Ambiental, Energía, Biome‐dicina y Agricultura (NANOTECH) Facultad de Ciencias Físicas Universidad Nacional Mayor de San Marcos Av. Venezuela Cdra 34 S/N Ciudad Universitaria Lima 15081 Perú; ^3^ Centro Brasileiro de Pesquisas Físicas (CBPF) R. Xavier Sigaud, 150 Urca Rio de Janeiro 22290‐180 Brazil; ^4^ Departamento de Física (DFis) Universidade Federal do Espírito Santo (Ufes) Av. Fernando Ferrari, 514 Vitoria 29075‐910 ES Brazil

**Keywords:** biosynthesis, functionalization, functionalized iron‐oxide, magnetic Fe‐oxide nanoparticles, polyphenols

## Abstract

By varying salt precursors and precipitating agents, polyphenol‐functionalized γ‐Fe_2_O_3_ nanoparticles (NPs) were systematically biosynthesized with controlled particle sizes and varying polyphenol layer thicknesses via two distinct approaches. In the *in situ* process (ISP), green tea (GT) extract influenced the formation of particles with different sizes during the synthesis, while in the after synthesis process (ASP), it enabled the functionalization of preformed γ‐Fe_2_O_3_ NPs. The use of GT extract significantly reduced the amount of precipitating agent (NH_4_OH or NaOH) commonly used in the coprecipitation method. However, even in a polyphenol‐rich environment, the Fe_3_O_4_ phase is detected only a few hours after the ISP. Results from various characterization techniques revealed that altering the GT extract content—*expressed* as percent weight‐to‐volume (x = %w/v)—affects the nanocrystallite size, magnetic behavior, and hyperfine properties, particularly in samples biosynthesized via ISP. Functionalization with GT extract enhanced the effective magnetic anisotropy of the γ‐Fe_2_O_3_ NPs compared to bare γ‐Fe_2_O_3_ NPs; however, this anisotropy decreased progressively as the x‐value increases. This trend suggests that the thicker organic layer reduced interparticle dipolar interactions by improving the dispersion of the magnetic NPs.

## Graphical abstract

Functionalized iron‐oxide nanoparticles exhibiting distinct physical characteristics (particle size and magnetic anisotropy) were successfully biosynthesized by modulating the precursor salts and using two different chemical synthesis pathways.

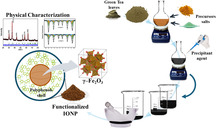

## 1 Introduction

Nanotechnology is a field of science that offers promising solutions to various issues.^[^ ^1^, ^2^, ^3^, ^4^, ^5^, ^6^, ^7^, ^8^ ^]^ Among the advances already achieved in this field are as follows: (i) the development of new technologies for storage information,^[^ ^1^, ^2^ ^]^ (ii) the targeted delivery of medicines to specific areas of the body,^[^ ^3^ ^]^ (iii) the creation of nanomaterials that improve nutrient absorption in plants,^[^ ^4^, ^5^ ^]^ and (iv) the adsorption of toxic metal ions in soil and water bodies contaminated by mining^[^ ^6^ ^]^ or wastewater.^[^ ^7^ ^]^ Most of these applications are typically carried out using Fe‐oxide‐based (IO) nanomaterials, specifically nanoparticles (NPs) of magnetite (Fe_3_O_4_), maghemite (γ‐Fe_2_O_3_), and hematite (α‐Fe_2_O_3_).^[^ ^3^, ^8^ ^]^ Chemical routes are the most commonly used to synthetize these types of IONPs^[^ ^3^, ^6^, ^7^, ^8^, ^9^, ^10^ ^]^ due to their relatively narrow particle size distributions (PSDs) and the reproducibility of the samples, which enables scale‐up processes.^[^ ^10^ ^]^ However, the synthesis protocols still need to be improved and/or modified to address current challenges and, of course, to generate distinct physical properties in the resulting nanomaterials. In this regard, it is well‐known^[^ ^3^, ^9^ ^]^ that the nanomaterial synthesis strongly depends on the chemical reaction conditions. Therefore, Fe‐based salts and precipitating agents used in coprecipitation reactions may play an important role in determining the final properties of the IONPs.

Besides improving the physical properties of IONPs—such as particle sizes and morphologies, dispersion in aqueous media, functionalization with polymers, and high surface‐to‐volume ratio—some of negative effects associated with their synthesis must also be mitigated. Specifically, the precipitating agent (e.g., NH_4_OH or NaOH), which controls the solution pH, must be optimized to reduce its usage in the final coprecipitation process; thereby lowering synthesis costs and minimizing environmental impact. Another issue that deserves special attention is that many NP applications require surface functionalization with organic phases,^[^ ^3^, ^6^, ^7^, ^8^, ^9^, ^10^ ^]^ a procedure that not only reduces NP agglomeration but may also enhance surface magnetic properties and chemical adsorptions.

Artificial polymers are often used in the functionalization process,^[^ ^11^, ^12^, ^13^ ^]^ but their use should either be avoided or significantly reduced. One alternative to address the issues associated with chemical routes is to take the advantage of organic components naturally found in the leaves of certain plants, thereby making the synthesis of IONPs more eco‐friendly. In this context, the use of leaf extracts has led to what is commonly referred to as “green synthesis”.^[^ ^12^, ^13^, ^14^ ^]^ As an example of such a process, we highlight the work by Chang et al.^[^ ^15^ ^]^ who used polysaccharide (starch and carboxymethylcellulose sodium) as stabilizing agents in the synthesis of magnetic IONPs.

Among natural organic phases, polyphenols—exclusively synthesized by plants—stand out as a rich source of antioxidants that combat free radicals.^[^ ^16^ ^]^ These molecules have the ability to form chelating structures with Fe‐ions, a condition essential for the coprecipitation route used in IONP synthesis.^[^ ^17^, ^18^ ^]^ Therefore, during the synthesis of IONPs, plant leaf extracts—rich in polyphenols—may create favorable conditions for reducing the amount of NH_4_OH or NaOH required as precipitating agents. Certain polyphenol components act as chelating agents with Fe‐ions, thereby lowering the necessary volume of precipitant in the synthesis process. Among the plant leaves that possess these properties, green tea (GT) leaves (*Camellia sinensis*) stand out. Green tea is of the most widely consumed beverages in the world and serves as a primary source of polyphenols, which comprise ≈36 % of the dried leaf content. In addition, green tea leaves contain carbohydrates (25%), proteins (15%), as well as smaller amounts of ash (5%), amino acids (4%), organic acids (1.5%), lipids (2%), and volatile substances (<1%), contributing to the complex composition of dried GT leaves.^[^ ^19^ ^]^ Moreover, the GT extract is rich in catechins, including (+)‐catechin (C), (–)‐epicatechin (EC), (–)‐gallocatechin (GC), (–)‐epigallocatechin (EGC), (–)‐catechin gallate (CG), (–)‐gallocatechin gallate (GCG), (–)‐epicatechin gallate (ECG), and (–)‐epigallocatechin gallate (EGCG), molecules that act as reducing agents for Fe‐ions.^[^ ^19^, ^20^ ^]^

Indeed, using *Camellia sinensis* in the synthesis of Fe‐based nanomaterials presents a promising green alternative to the conventional coprecipitation method, delivering both environmental advantages and enhanced material properties. This phytochemical‐mediated approach leverages the natural composition of green tea, particularly its high polyphenol content, which plays a dual role in the synthesis process: (i) acting as a reducing agent to facilitate the conversion of Fe‐ions and (ii) as a capping or stabilizing agent to prevent NP agglomeration. This dual function is critical for promoting, for instance, the formation of nanocrystalline IONPs with good magnetic behavior and enhanced colloidal stability. Compared to traditional synthetic routes, green tea‐mediated IONPs exhibit greater long‐term reactivity and structural integrity, making them effective in environmental applications.^[^ ^21^, ^22^ ^]^ Specifically, Fe‐based nanomaterials, synthesized using green tea or eucalyptus extracts, have demonstrated efficient nitrate removal from aqueous solutions, showing higher stability and sustained performance relative to chemically synthesized nanoscale zero‐valent iron (nZVI).^[^ ^21^ ^]^ Additionally, the nanomaterials synthesized by this green synthesis route have proven effective for the removal of various water contaminants (including toxic metals, dyes, chlorinated compounds, and pharmaceutical residues) highlighting their cost‐effectiveness and environmental compatibility.^[^ ^22^ ^]^

Therefore, in the present work, the formation of IONPs biosynthesized by a modified coprecipitation route in a polyphenol‐rich solution was carefully investigated. Different molar fractions of polyphenols, various iron salts (FeCl_2_/FeSO_4_•7H_2_O and FeCl_2_/FeCl_3_), and pH control agents (NH_4_OH or NaOH) were systematically used in two distinct biosynthesis routes—*in situ* synthesis process (ISP) and after synthesis process (ASP)—to assess their effects on the structural, morphological, hyperfine, and magnetic properties of the IONPs. An increasing in the molar fraction of polyphenols in the solution led a reduction of IONP size and enhanced surface functionalization, meaning the NPs were more effectively coated with organic compounds. This ensured successful functionalization of the IONPs surfaces and resulted in improved dispersion in aqueous medium when compared to our group's results using the conventional coprecipitation process.^[^ ^23^ ^]^

A comprehensive characterization of the obtained IONP samples was conducted using X‐ray diffraction (XRD), transmission electron microscopy (TEM), vibrating sample magnetometry (VSM), and ^57^Fe Mössbauer spectrometry. Thermogravimetric analysis (TGA) and Fourier transform infrared spectroscopy (FTIR) were also used to confirm the surface functionalization of the IONPs with GT components. The optimized biosynthesis process led to a reduction of ≈5 % in the amount of precipitating agent required to promote the NP nucleation and growth. This highlights the active role of GT extracts in the synthesis of IONPs, providing favorable conditions for NP formation. Depending on the iron salts used, variations in nanocrystallite sizes, particle morphology and magnetic anisotropy were observed. Specifically, the effective magnetic anisotropy increased compared to unfunctionalized $\gamma -Fe_{2}O_{3}\;$NPs but gradually reduces with higher GT extract content. These findings make a significant contribution to the literature by proposing a methodology for synthetizing IONPs with controlled size, morphology, dispersion, and different magnetic anisotropy. Consequently, this modified coprecipitation process demonstrates potential for scalability, as multiple similar samples were successfully reproduced with consistent properties here discussed.

## 2 Experimental Section

### 2.1 Materials

The GT extract was obtained from fresh green tea leaves that were carefully washed and naturally dried. With a blade mill, the dried leaves were first ground into a powder, which was then dissolved in ultrapure water for infusion. The solution for IONPs formation consisted of different molar fractions of GT extracts, along with ferrous sulfate heptahydrate (FeSO_4_•7H_2_O) and Fe chloride (FeCl_3_ and FeCl_2_) in an Fe^2+^/Fe^3+^ molar ratio of ≈0.5. Four series of IONPs were systematically biosynthesized and, for convenience, were named as Series‐A, B, C, and D (or simply Series‐j, with j = A, B, C, and D). Series‐A (ISP) and Series‐C (ASP) were synthetized with NH_4_OH (7–8 M) in a solution containing FeSO_4_•7H_2_O and FeCl_3_, while Series‐B (ISP) and Series‐D (ASP) were synthetized using NaOH (1 M) in a solution containing FeCl_3_•6H_2_O and FeCl_2_•4H_2_O. All chemical compounds, obtained from Sigma‐Aldrich, were used without further purification.

### 2.2 Preparation of the GT Extract

To take advantage of the polyphenols contained in the green tea leaf powders, different infusions were carefully prepared using 200 mL of ultrapure water and varying masses (*w*) of green tea powders. Each infusion was filtered with Whatman paper filter (≈11μm) and separated according to specific quantities for biosynthesis of the IONPs, i.e., concentrations of the GT extract in %w/v (weight/volume)—hereafter identified as GTx—were x = 1, 3, 5, 8, and 10.

### 2.3 Biosynthesis of γ‐Fe_2_O_3_ NPs Using the GT Extract: ISP

**Scheme 1:**
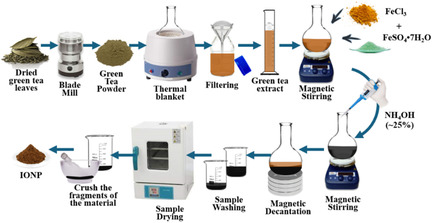
Schematic image of obtaining functionalized IONPs, progressing from GT extract to the final powdered nanomaterial of Series‐A samples, using a methodology similar to that. i described by Ramos‐Guivar et al.^[^ ^43^ ^]^

A volume of 100 mL of GT extract was magnetically stirred at 353 K and 900 revolution per minute (rpm) for 30 min. Considering an Fe^2+^/Fe^3+^ molar ratio of ≈0.5 and aiming for a final mass ≈4.0 g of IONPs, 5.2 g of FeSO_4_•7H_2_O and 6.0 g of FeCl_3_ were added to the solution for Series‐A or ≈9.3 g FeCl_3_•6H_2_O and ≈3.4 g FeCl_2_•4H_2_O for Series‐B. Subsequently, the stirring speed was gradually increased to 1200 rpm. After 30 min, 5 mL of NH_4_OH (7–8 M) were slowly added to the Series‐A solution [or 49 mL NaOH (1 M) for Series‐B], immediately triggering a color change to blackish, visually indicating the formation of IONPs. The magnetic properties of the sample magnetism were confirmed bringing a neodymium permanent magnet close to the flat‐bottomed round ball. The samples were then carefully washed with distilled water until the pH was adjusted to ≈7. Each powder sample was dried in a muffle furnace at 333 K for 24 h. For convenience, samples exhibiting core‐shell features were designated as A‐NP@GTx for Series‐A and B‐NP@GTx for Series‐B (with x = 1, 3, 5, 8, and 10). The synthesis process (ISP) for Series‐A (FeSO_4_/FeCl_3_ and NH_4_OH) is graphically represented in **Scheme** **1**. It is important to note that the Series‐A and Series‐B samples were synthetized in replicates and showed consistent results, indicating that the GT extract‐based biosynthesis method is scalable and holds potential applications where large quantities of IONPs are required.

### 2.4 Functionalization of γ‐Fe_2_O_3_ NPs Using the GT Extract: ASP

**Scheme 2:**
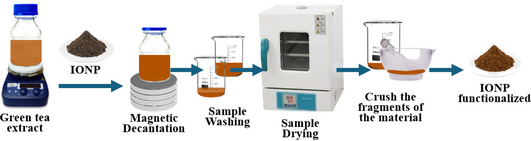
Schematic image of the process of obtaining Fe‐oxide functionalized with GT extract. In this case, the GT extract was kept in vigorous agitation at 300 K, and then, the IONPs were added. The recurrent process of magnetic decanting, washing of the sample with distilled water, drying, and subsequent crushing of the sample obtained is continued.

The functionalization of 4 g of γ‐Fe_2_O_3_ NPs, previously synthetized according to the procedures reported in the literature,^[^ ^23^, ^24^ ^]^ was also carried out. γ‐Fe_2_O_3_ NPs with average sizes of 12–13 nm were obtained using FeSO_4_/FeCl_3_ and NH_4_OH, while thinner IONPs (8–9 nm) were formed via the reaction with FeCl_2_/FeCl_3_ and NaOH. These results may indirectly suggest that the smaller IONPs are formed when FeCl_2_/FeCl_3_ and 1 M NaOH are used, as the results were reproducible. Returning to the functionalization in the ASP, the previous synthetized IONPs were mixed at 300 K with 100 mL of GT extract at x = 5, 8, and 10, as illustrated in **Scheme** **2**. The solution was kept at 300 K under stirring at 1200 rpm and then dried at 333 K for 24 h. Once again, the resulting nanomaterials were labeled as follows: Series‐C (C‐NP@GTx) for those synthetized with FeSO_4_/FeCl_3_ and NH_4_OH and Series‐D (D‐NP@GTx) for those obtained with FeCl_3_•6H_2_O/FeCl_2_•4H_2_O and NaOH.

### 2.5 Structural Properties: XRD Measurements

XRD experiments for all series were performed using a RIGAKU Ultima IV diffractometer equipped with a Cu K_α_ radiation source (λ = 1.5418 Å), generated by a Cu anode operating at 30 mA and 40 kV under laboratory ambient conditions. XRD data were collected over a 2*θ* range from 20° and 80°, with a step size of Δ2*θ* = 0.02° and a counting time of 15 s per step, using a Bragg–Brentano geometry, commonly applied to polycrystalline samples. Crystallographic information file #9 006 316 (retrieved from the crystallographic database^[^ ^25^ ^]^) was identified using the Match! v3 software.^[^ ^26^ ^]^ Rietveld refinements of the XRD diffractograms were performed using the free software Fullprof (version January 2021). A standard CeO_2_ sample was used to determine the instrumental line broadening, improving the accuracy of mean nanocrystallite estimations for the IONPs. The Cagliotti parameters used for the peak profile fitting were U = 0.003, V = −0.001, and W = 0.008.

### 2.6 Thermal Stability of the Sample Phases: Thermogravimetric Measurements

Thermogravimetric analyses (TGA) of all samples were performed using a Shimadzu TGA‐50 instrument (Shimadzu Corporation, Kyoto/Japan). The measurements were carried out under an argon flow rate of 50 mL min^−1^, with a heating rate of 10 °C min^−1^, over a temperature range from 300 to 1200 K.

### 2.7 Morphological Properties: TEM

PSD and morphology of the most representative samples from Series‐A, B, C, and D were analyzed using a JEOL 2100 F transmission electron microscope (TEM) operating at 200 kV (JEOL Ltd., Tokyo, Japan), in both conventional transmission and high‐resolution modes. PSD data were obtained by measuring ≈500 particles from 30 to 35 micrographs. The resulting histogram‐like plots were fitted using a log‐normal distribution method, following the procedure described in the literature.^[^ ^27^ ^]^ Polydispersity values were calculated based on the standard deviation of the fitted log‐normal distribution.

### 2.8 FTIR Analysis

Selected samples from all series were analyzed by FTIR spectroscopy using a Bruker Vertex 70v spectrometer (Bruker Optik, Ettlingen, Germany). Measurements were performed in absorbance mode, with the sample chamber maintained under a vacuum of ≈10^−2^ Torr to minimize interference from atmospheric gases. The samples were prepared as KBr pellets to ensure optimal infrared transmission, and the spectra were recorded at a resolution of 4 cm^−1^ over the spectral range of 400 to 4000 cm^−1^.

### 2.9 Magnetic Properties: Vibrating Sample Magnetometer (VSM)

Isotherm magnetization versus applied field (*M*(*H*)) curves were recorded using a PPMS‐Evercool II setup (Quantum Design Inc., USA) equipped with a VSM. Measurements were carried out for all samples from the four series at both 300 K and 4 K, over a magnetic field range of ±5 T, sufficient to reach saturation magnetization (*M*_S_). The values of *M*_S_, coercive field (*H*_C_), and effective anisotropy (*K*_eff_) parameters were numerically determined by fittings the high‐field region (20–50 kOe) of the *M*(*H*) curves using the Law of Approach to Saturation (LAS) model, as described in the literature.^[^ ^23^ ^]^

### 2.10 Hyperfine Properties: ^57^Fe Mössbauer Spectrometry

A helium closed‐cycle cryostat from Janis Research Company *Inc*. (USA) was used to acquire transmission ^57^Fe Mössbauer spectra at both 300 K and 15 K for all series samples. A 25 mCi ^57^Co:**Rh** radioactive source, maintained at 300 K throughout the experiments, was used. The spectrometer (excluding the electronics) was mounted on an antivibration table, and the drive system (coupled to ^57^Co:**Rh**) operated in sinusoidal mode to produce the Doppler shift required for Mössbauer spectroscopy. The absorbers were placed in nylon holders, with effective thicknesses adjusted to correspond to ≈0.1 mg of ^57^Fe per cm^2^. The ^57^Fe Mössbauer spectra were fitted using the Mosswinn 4.0i software.^[^ ^28^ ^]^ The isomer shift (*IS*) values are reported relative to α‐Fe at 300 K.

## 3 Results and Discussion

### 3.1 Structural Properties: XRD Analysis

**Figure 1:**
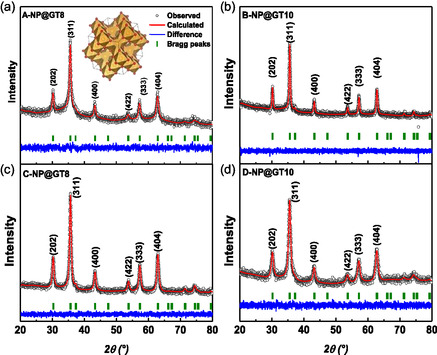
XRD diffractograms for selected j‐NP@GT‐x samples, where j‐ and x‐values are indicated. The (*hkl*) Miller planes for the space group are displayed. The red full lines represent the calculated diffractograms obtained using the Thompson‐Cox‐Hastings (TCHX) function, while the black points are the experimental data, and the blue lines are the differences between the observed and calculated intensities.

The refined XRD diffractograms of selected samples from the four series are shown in **Figure** **1** (additional XRD diffractograms are provided in Figure S1, Supporting Information). All XRD diffractograms were successfully refined using only the $\text{Fd}\bar{3}m$ space group, corresponding to the γ‐Fe_2_O_3_ crystallographic phase. In other words, given the negligible differences between experimental and calculated XRD diffractograms (blue lines shown in Figure 1), it can be concluded that all samples exhibit a single crystalline phase, specifically, the cubic inverse spinel commonly observed in both Fe_3_O_4_ and γ‐Fe_2_O_3_ NPs.^[^ ^29^ ^]^ The organic phases coating the NP surfaces are X‐Ray amorphous, and thus not detectable in the diffractograms. The refined structural parameters are summarized in **Table** **1** and Table S1, Supporting Information.

**Table 1:** Highlighted crystallographic and morphology parameters obtained for selected samples of the Series‐j (j = A, B, C, and D). Nanocrystalline sizes obtained from XRD (*t*_XRD_), mean particle sizes (*t*_TEM_) from TEM and the cubic lattice parameter of the spinel structure.

Samples	*t*_XRD_ ± 1 [nm]	*t*_TEM_ [nm]	Lattice parameter of cubic structure ± 0.0001 [nm]
A‐NP@GT8	9	8 ± 4	0.8365
B‐NP@GT10	23	26 ± 5	0.8364
C‐NP@GT8	13	14 ± 4	0.8356
D‐NP@GT10	8	13 ± 4	0.8371

**Figure 2:**
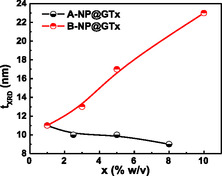
Behavior of crystallite size (*t*_XRD_) as a function of the GT extract content (x) in the Series‐A (black symbols) and Series‐B (red symbols) samples.

The calculated lattice parameters are ≈0.836 nm (Table 1), a value commonly observed in γ‐Fe_2_O_3_ NPs.^[^ ^30^ ^]^ On the other hand, the two synthesis routes *—* ASP and ISP *—* exhibited different trends in mean nanocrystallite sizes. In Series‐A, the average crystallite size decreased as the GT extract content (x) increased, whereas in Series‐B, the crystallite sizes gradually increased with increasing x, as shown in **Figure** **2**. However, as presented in Table 1, the nanocrystallite sizes of Series‐B and D samples *—*synthetized with same precursors (FeCl_2_/FeCl_3_), precipitating agent (NaOH), and lower molarity (1 M) *—* show opposite trends when compared to Series‐A and Series‐C samples, which were synthetized with equal salts (FeSO_4_/FeCl_3_), precipitating agent (NH_4_OH), and higher molarity (7–8 M). These XRD‐derived nanocrystallite results (*t*_XRD_) indicate that not only the precursor salts, precipitating agents, and molarities influence the formation of the γ‐Fe_2_O_3_ NPs, but the GT extract also plays a significant role in the synthesis process. Since XRD does not provide information about amorphous organic phases or surface functionalization, the possible layer polyphenol thickness organic (*t*_org_) will be discussed on the TGA results.

### 3.2 Morphological Properties: TEM Analysis

**Figure 3:**
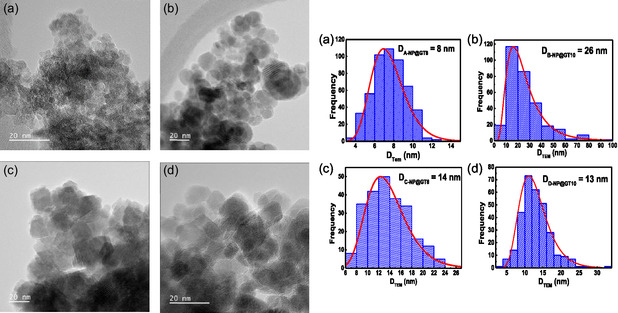
(left panel) TEM images and (right panel) PSD curves of the a) A‐NP@GT8, b) B‐NP@GT10, c) C‐NP@GT8, and d) D‐NP@GT10 samples. The PSD curves were built taking 300 particles from different images and fitting the histogram graphics with lognormal function.

**Figure** **3** displays the TEM images (left‐panel) obtained for selected samples from Series‐j (j = A, B, C, and D). Additional TEM images are provided in Figure S2, Supporting Information. As shown in Figure 3, the individual particles from Series‐A and Series‐B exhibit predominantly circular‐like shapes, whereas Series‐C and Series‐D present additional polyhedral‐like crystal morphologies. In both cases, the TEM images revealed well‐defined crystalline planes, indicating that the IONPs are well‐structured. Amorphous‐like features are also observed on the surfaces of the NPs. These features are likely associated with the presence of organic‐type compounds used in both synthesis routes (ISP and ASP). Such organic phases appear to suppress the agglomeration typically observed in bare γ‐Fe_2_O_3_ NPs synthetized via conventional coprecipitation process. This effect is particularly pronounced in the ASP samples, where the 13 nm thick γ‐Fe_2_O_3_ NPs, frequently reported by our group using the traditional coprecipitation method,^[^ ^31^ ^]^ now appear better dispersed, likely due to the presence of an organic surface layer (*t*_org_), which increases with x, as will be discussed later using TGA data. Furthermore, the mean particle sizes (*t*_TEM_), obtained from PSD analyses, are in full agreement with the average crystallite sizes (*t*_XRD_) determined from XRD data, showing a decreasing trend as × increases (see Table 1). The close agreement between $t_{\text{TEM}}\text{ and }t_{\text{XRD}}$ values suggests that the γ‐Fe_2_O_3_ NPs behave as single‐crystalline domains. The particle size range of all IONPs also suggest for a single magnetic domain behavior.

To summarize the structural and morphological properties of the γ‐Fe_2_O_3_ NPs, particularly those from Series‐A and Series‐B samples (i.e., ISP), the organic compounds derived from the GT extract do not appear to form an effective barrier to NP growth. In Series‐A, a decrease in the crystallite size ($t_{\text{XRD}}$) is observed with increasing x‐values, whereas in Series‐B, an opposite trend is noted, with $t_{\text{XRD}}$ increasing as the x‐parameter increases. These results indicate that the γ‐Fe_2_O_3_ NPs are within the nanometric size regime, exhibit single crystalline and single magnetic domain behavior, and are capped by organic layers, an observation that will be further supported by Mössbauer, FTIR, and TGA data. The assumption of a single magnetic domain behavior is based on the fact that the typical domain‐wall size range for the γ‐Fe_2_O_3_ phase (≈20−40nm)^[^ ^32^ ^]^ is larger than the $t_{\text{XRD}}$ values (≈9−20nm) obtained in this work.

### 3.3 FTIR Analysis

The presence of phenolic compounds in GT extracts, typically associated with hydroxyl groups and aromatic rings, appears in the high wavenumber regions (*k* > 1100 cm^−1^).^[^ ^33^, ^34^ ^]^ In contrast, the functional groups related to Fe‐oxides are generally observed in the low wavenumber region (*k* < 900 cm^−1^).^[^ ^35^ ^]^ For comparison, an FTIR experiment of the pure GT extract was recorded and is displayed in Figure S3a, Supporting Information. This FTIR spectrum serves as a reference sample for identifying organic phases, but it was not possible to resolve contributions from individual phenolic compounds—as those described in the introduction—due to limitations in the experimental resolution. The FTIR spectrum of the GT extract can be divided in two main regions: Region‐I (3700 < *k* < 2750 cm^−1^) and Region‐II (1750 < *k* < 1450 cm^−1^). Region‐I contains two major bands, one centered around 3390 cm^−1^ (yellow marked), which is attributed to O—H stretching modes from alcohols as well as N—H stretching from amine groups. These functionalities are likely present in the polyphenolic constituents of the GT extract and may also arise from residual water absorbed from the environment. Additionally, O—H stretching from hydroxyl groups on the surface of iron‐oxides may contribute to this band, reflecting interaction between the inorganic matrix and the organic coating. In Region‐II, the band near 1616 cm^−1^ (highlighted in green) corresponds to C=C stretching vibrations of aromatic rings in polyphenols.^[^ ^36^ ^]^ Other notable features in this region include bands at ≈1370 cm^−1^ and 1080 cm^−1^ (dashed vertical lines), which are attributed to C—O stretching and O—H bending vibrations (both in‐plane and out‐of‐plane).^[^ ^37^ ^]^

**Figure 4:**
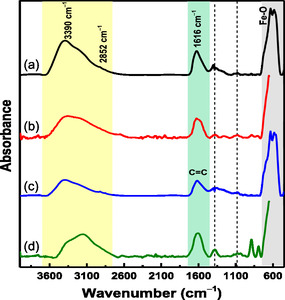
300 K FTIR spectra for the a) A‐NP@GT5, b) B‐NP@GT5, c) C‐NP@GT5, and d) D‐NP@GT5 samples. The vertical yellow and green colored strips are associated with C—O and O—H bonds, while the band associated with Fe‐oxide bonds is shown in gray. Dashed vertical lines are attributed to C—O stretching and O—H bending vibrations.

FTIR spectra of selected samples from all series as well as specific spectra of the A‐NP@GTx and C‐NP@GTx samples, with varying x‐values, are shown in **Figure** **4** and Figure S3b,c (Supporting Information), respectively.

From Figure S3b,c, Supporting Information, it can be observed that the intensity of the bands associated with phenolic compounds increases with the x‐value. In other words, these signals are more prominent in the A‐NP@GT8 sample compared to A‐NP@GT1. This trend suggests that the GT extract effectively functionalized the surface of the γ‐Fe_2_O_3_ NPs. Specifically, the bands attributed to the Fe‐oxide phase appear in the range 445–735 cm^−1^,^[^ ^7^, ^38^ ^]^ as highlighted in Figure 4 (gray‐shaded region). The FTIR spectra of the C‐NP@GTx samples (Figure S3c, Supporting Information) exhibit similar spectral features to those of the A‐NP@GT8 sample, indicating that the presence of catechins contributes to the formation of an organic coating layer on the IONP surfaces. Therefore, it can be inferred that the functionalized γ‐Fe_2_O_3_@GT(*t*_org_) NPs possess an organic layer, whose thickness (*t*_org_) increases with the x‐values. This organic layer effectively separates γ‐Fe_2_O_3_ NPs, reducing their dipole–dipole magnetic interactions commonly observed in unfunctionalized γ‐Fe_2_O_3_ NPs synthesized by conventional coprecipitation method. The final size of the γ‐Fe_2_O_3_@GT(*t*_org_) NPs is influenced both by the chemical precursors used and by the synthesis routes (ISP or ASP), with the ISP process appearing to be more effective (a thicker layer). This conclusion is further supported by TGA data, which will be discussed ahead.

### 3.4 Hyperfine Properties: ^57^Fe Mossbauer Analysis

**Figure 5:**
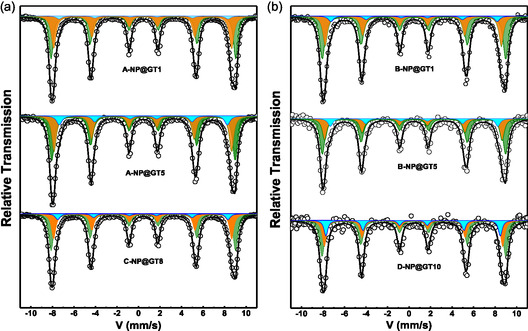
15 K ^57^Fe Mössbauer spectra of the j‐NP@GT‐x nanohybrids, with j and x‐values displayed in the figure. Black full lines are due to the fittings using the three components: two from the spinel (A‐orange and B‐green) and the third one (cyan) due to Fe^3+^ ions functionalized with organic phases of the GT‐extract. j = A and B are samples by ISP, while C and D, are by ASP.

The 15 K ^57^Fe Mössbauer spectra recorded for selected samples from all series are displayed in **Figure** **5**, while the spectrum at 15 K for a bare γ‐Fe_2_O_3_ from Series‐C (used as a reference sample) is presented in Figure S4, Supporting Information. All spectra recorded at 15 K exhibit six asymmetric absorption lines, which are characteristic of Zeeman splitting resulting from a magnetically ordered state of Fe‐ions occupying at least two distinct crystallographic sites within the spinel cubic structure, a crystalline phase also confirmed by XRD analysis. To fit the 15 K Mössbauer spectra of the functionalized γ‐Fe_2_O_3_@GT(*t*_org_) NPs, the following conditions were adopted: (i) the Mössbauer spectrum of the bare γ‐Fe_2_O_3_ NPs (Figure S4, Supporting Information) was well fitted using two sextets corresponding to Fe^3+^ ions at the tetrahedral (A) and octahedral (B) sites of the spinel structure, with site population of 33% for A‐site and 67% for B‐site; (ii) the broader absorption linewidth associated with the B‐site in the bare γ‐Fe_2_O_3_ NPs was attributed to higher concentration of octahedral sites at or near the particle surface, as previously reported in the literature;^[^ ^39^ ^]^ and (iii) additional asymmetries in the line shapes of the Mössbauer spectra were consistently observed in functionalized samples compared to the bare γ‐Fe_2_O_3_ NPs (reference sample), indicating chemical interactions at the particle surface. As a result, the Mössbauer spectra at 15 K for the functionalized samples were fitted using three components: the two sextets identified in the bare γ‐Fe_2_O_3_ NPs (Figure S4, Supporting Information) plus a 3rd component (depicted by the cyan subspectrum) attributed preferentially to Fe^3+^ ions at octahedral sites that interact chemically with polyphenols at the NP surfaces. All hyperfine parameters are displayed in Table S2a,b, Supporting Information. Once more, the presence of three components is supported by the observation that the asymmetric line profiles found in the γ‐Fe_2_O_3_@GT(*t*_org_) NPs are not fully resolved, unlike those in pure γ‐Fe_2_O_3_ NPs, which typically display a relative absorption area ratio of 5:3 for B‐to A‐sites.^[^ ^30^, ^31^ ^]^ When analyzing the IS values, only hyperfine parameters consistent with Fe^3+^ spin states were observed, confirming the exclusive presence of Fe^3+^ in the γ‐Fe_2_O_3_ phase.^[^ ^27^ ^]^ Therefore, it can be definitively stated that no Fe_3_O_4_ (magnetite) phase was formed within a few days after the biosynthesis via either ISP or ASP methods. This conclusion is further supported by the magnetization data, which will be discussed in a subsequent section. Additionally, the relative fraction of the 3rd‐spectral component appears to increase with the x‐value, suggesting that the interaction between Fe^3+^ ions at octahedral sites and polyphenols become more significant as the GT extract concentration increases during the synthesis. To elucidate precisely this assumption, only in‐field ^57^Fe Mössbauer experiments could help, as shown in the literature for GT‐Fe‐based nanomaterial,^[^ ^22^ ^]^ but we are not able to perform it at this moment.

The 300 K Mössbauer spectra, displayed in Figure S5, Supporting Information, are more complex than those recorded at 15 K. In general, the 300 K spectra present broader absorption lines with a reduced asymmetry, and in some cases, the presence of a central doublet is observed. This doublet is attributed to very small NPs exhibiting superparamagnetic (SPM) behavior at 300 K. These results are consistent with the broad PSD observed in TEM images (Figure 3 and Figure S2a,b, Supporting Information), which reveal γ‐Fe_2_O_3_ NPs with average sizes in the 4–8 nm range, commonly associated with the SPM regime at 300 K. For fitting purposes, the 300 K spectra were numerically modeled using a magnetic hyperfine field distribution (MHFD) component, and when necessary, an additional doublet. Both components (MHFD and doublet) exhibited similar average isomer shift ⟨IS⟩ values of ≈0.36 mm s^−1^, a characteristic value for Fe^3+^ ions (see Table S2a,b, Supporting Information). Importantly, no spectral signatures of Fe^2+^ ions were detected, reinforcing the conclusion that no Fe_3_O_4_ phase was formed, even after the surface functionalization with organic compounds.

### 3.5 Magnetic Properties: VSM Analysis

**Figure 6:**
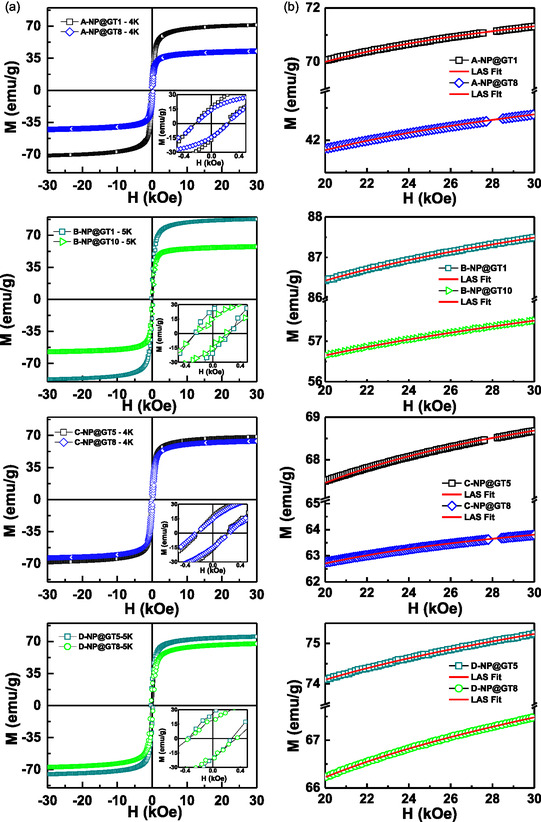
4 K *M*(*H*) loops in a) and their fittings with the LAS model in b) for sample of all series (j‐NP@Gt‐x) with different j and x‐values, as indicated.

**Figure 7:**
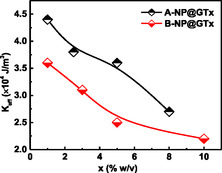
The behavior of the effective magnetic anisotropy (*K*_eff_), calculated at 4 K, as a function of the GT‐extract content (x) in the Series‐A and Series‐B samples.

The 4 K *M*(*H*) loops of selected samples from the four series are presented in Figure 6, with x‐values indicated in the figure. The Inset in panel (a; left) show a zoomed view of the low‐field region, clearly revealing the presence of coercive fields (*H*_*C*_) typically associated with ferro‐ or ferrimagnetic behavior, as expected for γ‐Fe_2_O_3_ NPs. To quantify the magnetic properties of the samples, the LAS model^[^ ^23^ ^]^ was applied to fit the high‐field portion of the *M*(*H*) curves. These fittings are represented by red lines in panel (b, right) of **Figure** **6**. The main magnetic parameters extracted from the fittings (*M*_S_, *H*_C_ and *K*_eff_ ) are summarized in **Figure** **7** and Table S3, Supporting Information. It is important to note that *M*_S_ value of γ‐Fe_2_O_3_ NPs is highly sensitive to several factors, including oxidation state, particle size, synthesis, functionalization, and degree of agglomeration. For NPs synthesized via coprecipitation, the reported *M*_S_ values at 4 K typically range between 60 and 78 emu g^−1^ at 4 K, depending primarily on particle sizes.^[^ ^40^ ^]^ Accordingly, the *M*_S_ values for both ISP and ASP samples in this study fall within this expected range (See Table S3, Supporting Information), except the B‐NP@GT1 sample (the explanation can be found at the end of the section).

The data also indicates that the *M*_S_ values gradually decrease with increasing x‐values in all samples. This trend suggests a higher concentration of organic‐type compounds on the NP surface at larger x, which contribute non‐magnetically, and thus reduce the net magnetization. For instance, when comparing Series‐A and Series‐C samples—both synthesized using the same precipitating agent–NH_4_OH—but with different x‐values, the C‐NP@GT8 sample exhibits a larger particle size (≈14 nm) yet a lower *M*_S_ value compared to the A‐NP@GT1 sample, which has a smaller particle size (≈10 nm) and a higher *M*_S_ value. This behavior supports the hypothesis that increased surface functionalization with polyphenols dampens the magnetic response. Additionally, TGA data, to be discussed in the next section, revealed a higher weight loss (WL) for the C‐NP@GT8 sample, further confirming the presence of a thicker organic layer.

Effective anisotropy constants (*K*_eff_) were also calculated using the LAS model. For bare γ‐Fe_2_O_3_ NPs from Series‐C to Series‐D (prior to functionalization), a *K*_eff_ value of ≈1.9 × 10^4^ J m^−3^ was obtained. This is at least one order of magnitude higher than the bulk value for γ‐Fe_2_O_3_, which is 4.7 × 10^3^ J m^−3^^[^ ^41^ ^]^ and is consistent with the enhanced surface and finite‐size effects expected at the nanoscale. Figure 7 illustrates the behaviors of *K*_eff_ at 4 K as a function of x‐value for the ISP samples of Series‐A and Series‐B. A gradual reduction in *K*_eff_ is observed as the x‐value increases, a trend that parallels the decrease in *M*_S_, and also suggests that the increased organic surface coverage suppresses magnetic anisotropy. Moreover, *K*_eff_ values were also calculated at different temperatures and showed a decreasing trend with increasing temperature, consistent with the behavior typically observed in conventional ferromagnetic materials.

Regarding IONPs discussed in this work, two main conclusions can be drawn. First, functionalization with organic phases leads to an increase in the *K*_eff_ values. For example, *K*_eff_ increases from 1.9 × 10^4 ^J m^−3^ for bare γ‐Fe_2_O_3_ NPs to ≈4.4 × 10^4 ^J m^−3^ for the A‐NP@GT‐1. Second, when comparing Series‐A and Series‐B, samples with larger particle sizes (i.e., Series‐B) tend to exhibit lower *K*_eff_ values. This indicates that larger NPs contribute less to surface anisotropy, due to a reduced surface‐to‐volume ratio. Additionally, for both series, a gradual decrease in *K*_eff_ with increasing x‐value is observed. This trend can be primarily attributed to a reduction in interparticle dipole–dipolar interactions, caused by a thicker organic layer (*t*_org_) formed by the polyphenol's compounds on the NP surface. In other words, as the x‐value increases, the organic coating grows thicker, spacing out the particles and diminishing dipolar magnetic coupling (i.e., reducing IONP‐*t*_org_‐IONP interactions). Thus, although the crystallite size (*t*_XRD_) behavior differs between Series‐A and Series‐B (as shown in Figure 2), the *K*_eff_ values (Figure 7) follow a similar decreasing trend with x. Still, the magnitude of *K*_eff_ appears to be primarily influenced by particle size, which is ultimately determined by the chemical precursors and precipitating agents used in the ISP process. Before concluding this section, it is worth mentioning that some Series‐B samples initially exhibited *M*_S_ values as high as ≈87 emu g^−1^, exceeding the typical value for bulk γ‐Fe_2_O_3_. This observation suggests the temporary presence of the Fe_3_O_4_ phase in freshly synthesized NPs. However, despite the organic layer stabilizing the surfaces, the magnetization values dropped to those characteristics of γ‐Fe_2_O_3_ NPs, just a few hours after synthesis, indicating oxidation of the Fe_3O_4 NPs. Further insights into the thermal stability of the functionalized γ‐Fe_2_O_3_ NPs will be obtained through magnetization measurements conducted after TGA experiments.

### 3.6 Thermal Stability: TGA Analysis

**Figure 8:**
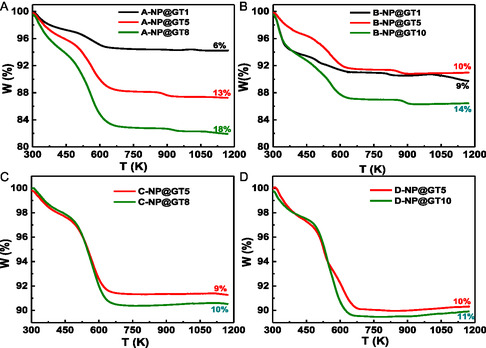
WL (W(%)) versus temperature for the ISP (top—Series‐A and Series‐B) and for the ASP (bottom—Series‐C and Series‐D) samples.

Thermal stability at elevated temperatures of the functionalized γ‐Fe_2_O_3_ NPs (j‐NP@GT‐x nanohybrids) was initially investigated through TGA analysis for all samples. The TGA curves, along their respective derivatives, for selected samples are presented in **Figure** **8** and Figure S6, Supporting Information, respectively. First, the ISP samples (Series‐A and Series‐B) exhibit a clear increase in WL percentage [W(%)] that roughly scales with increase x‐value, i.e., samples synthesized with higher GT extract concentrations tend to show higher WL. Among these, Series‐A consistently presents higher WL value than Series‐B samples. This observation supports the notation that Series‐B samples, having larger particles sizes, exhibit a lower surface area‐to‐volume ratio and, consequently, a smaller relative mass of surface‐bound organic materials compared to the smaller‐sized NPs of Series‐A. In contrast, the ASP samples (Series‐C and Series‐D) also show WL trends that scale with increasing x‐value; however, the absolute WL values in ASP are significantly lower than those observed in the ISP samples for equivalent x‐values. This result strongly indicates that the ISP process is more efficient in promoting surface functionalization with polyphenolic compounds that the ASP route. Taken together, these findings suggest that a higher fraction of surface‐bound organic compounds—specially in ISP samples—facilitates greater NP dispersion by reducing interparticle dipole–dipole interactions, consistent with the magnetic behavior previously discussed. Therefore, ISP emerges as the more effective strategy for obtaining well‐dispersed, functionally stabilized γ‐Fe_2_O_3_ NPs.

From the derivative TGA curves (Figure S6, Supporting Information), three distinct peaks are observed in the ISP samples, while only two are clearly identifiable in the ASP samples. The first peak, located below 450 K, and the second, around 600 K, are common to both sample types. These are attributed to the loss of volatile substances from the NP surfaces, specifically, adsorbed water (below 450 K) and polyphenolic compounds from the GT extract (near 600 K). Notably, the area of the 600 K peak increases with the x‐value, indicating a greater mass of organic material present at higher GT extract concentrations. A third peak, observed well above 600 K, is clearly visible only in some ISP samples, and is associated with the γ‐Fe_2_O_3_ to α‐Fe_2_O_3_ phase transition.^[^ ^42^ ^]^ Interesting, this γ→α phase transition is not prominently detected in the ASP samples at the expected temperature of about 910 K. Instead, a broad knee around 1200 K in the derivative curves of the C‐NP@GTx samples suggests that the transition may have been shifted to higher temperatures, potentially delayed by the presence of the organic coating. This behavior implies that the organic functionalization layer in ASP samples may be more thermally stabilizing, possibly inhibiting or delaying the structural transformation of the Fe‐oxide phase up to maximum temperature of the TGA setup (1200 K).

To gain a better understanding of the thermal stability of the samples after reduction of volatile substances, as revealed by the TGA experiments, the B‐NP@GTx samples (ISP) were selected for further investigation, as these were the ones where that three distinct peaks in the TGA curves were observed. Thus, *M*(*H*) loops at 4 K were recorded for samples annealed under three thermal conditions, all starting from 300 K: (i) Range‐I: annealing up to 403 K, (ii) Range‐II: annealing up to 600 K, and (iii) Range‐III: annealing up to 910 K. Stated otherwise, these temperature intervals were chosen based on the characteristic features observed in the TGA derivative curves: Range‐I and Range‐II correspond to the two main WL peaks (removal of adsorbed water and polyphenols), while Range‐III corresponds to the temperature region where the γ→α structural transition is expected to occur.

**Figure 9:**
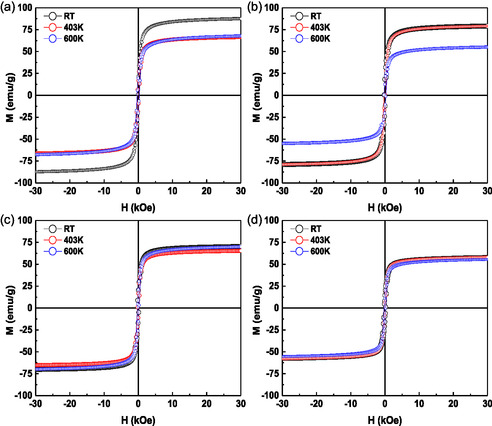
4 K *M*(*H*) loops of the B‐NP@GT‐x samples (x‐values are a) 1, b) 3, c) 5, and d) 10). Red symbols are for samples annealed up to 403 K, while the blue ones are for the samples annealed up to 600 K. In both annealing ranges, a new sample was measured.

The 4 K *M*(*H*) loops for the annealed and unannealed (RT) samples are displayed in **Figure** **9** (for Range‐I and Range‐II) and Figure S7, Supporting Information (for Range‐III). For comparison, the *M*(*H*) curves of unannealed materials (RT samples measured at 4 K few days after synthesis) are also included in Figure 9. These unannealed samples already exhibit *M*_S_ values in the range typically associated with γ‐Fe_2_O_3_ NPs (60–78 emu g^−1^). Interestingly, the *M*_S_ values remain nearly unchanged for most samples annealed up to 403 K. This suggests that, while the majority of volatile contents has minimal impact on the magnetic phase at lower temperatures, the x = 1 sample (B‐NP@GT‐1) likely contained residual Fe_3_O_4_ or core‐shell Fe_3_O_4_/γ‐Fe_2_O_3_ structures (see Figure 9a), which initially exhibited *M*_S_ value of ≈87 emu g^−1^, as discussed above (this value is consistent with the presence of Fe_3_O_4_, known for its higher magnetization compared to γ‐Fe_2_O_3_). However, over time and/or upon heating (even modestly), the *M*_S_ value of ≈87 emu g^−1^ decreases to those characteristics of the γ‐Fe_2_O_3_ NPs (60–78 emu g^−1^), which is consistent with the progressive oxidation of Fe_3_O_4_ to γ‐Fe_2_O_3_, as discussed above. Expressing differently, this effect is clearly illustrated in the magnetization curve measured immediately after the synthesis for the x = 1 sample (see Figure 9a), highlighting the importance of thermal and temporal stability in maintaining or transforming the magnetic phases.

Moreover, considering the WL‐value up to 600 K and no substantial changes on *M*_S_ values of the B‐NP@GT‐5 (Figure 9c) and B‐NP@GT‐10 (Figure 9d) samples, it can be assumed that the IONPs of these hybrid samples are still covered (total or partially) with the organic phases from the GT extract. In other words, considering the TGA and *M*(*H*) results, it can be supposed that at higher x‐values (5 and 10), there is a thicker organic layer before annealing, which was subsequently volatilized partially when the sample temperature increased to 600 K. This indicates that γ‐Fe_2_O_3_@GT(*t*_org_) NPs are thermically stable for temperatures up to 600 K. It can be also inferred that there is a porosity (holes) on the organic layer that favors oxygen migration leading to the observed conversion of Fe_3_O_4_ into γ‐Fe_2_O_3_, as shown in the B‐NP@GT‐1 sample (in bare IONPs, the oxidation practically occurs instantaneously after the synthesis).

At and above 900 K (see Figure S7, Supporting Information), the magnetization of annealed samples drastically reduces to values smaller than 3 emu g^−1^ at 4 K. However, the *M*(*H*) loops still show two magnetic contributions: a hysteretic one associated with residual γ‐Fe_2_O_3_ phase plus a contribution that increases roughly linear with applied field, indicating an antiferromagnetic phase, found, e.g., in α‐Fe_2_O_3_ NPs. Thus, this observation indicates that the γ‐Fe_2_O_3_ NPs has been partially transformed to α‐Fe_2_O_3_ NPs for temperatures up 910 K, i.e., forming IONPs with core‐shell features such as γ‐Fe_2_O_3_@α‐Fe_2_O_3_, as shown by the two magnetic components of Mössbauer spectrum displayed in Figure S8, Supporting Information, for the sample annealed at 910 K. On the other hand, a pure hematite (α‐Fe_2_O_3_) phase was found in the spectrum recorded at 1173 K (it can be assumed that at 900 K all organic compounds are volatilized), a result that agrees with the TGA data.

In summary, our results indicate that the polyphenols from the GT extract enhance the magnetic properties and thermal stability of the functionalized IONPs, in close agreement with previous reports on iron NPs (GT‐Fe) and SPM iron oxide (sm‐GT) also synthesized using GT extract, which demonstrated good physical properties^[^ ^21^, ^22^ ^]^ and biocompatibility for environmental remediation.^[^ ^21^ ^]^ Thus, this green synthesis approach offers a cost‐effective, sustainable, and efficient method for producing functionalized nanomaterials applicable to pollutant treatment.

## 4 Conclusion

Functionalized γ‐Fe_2_O_3_ NPs with varying polyphenol layer thicknesses [γ‐Fe_2_O_3_@GT(*t*_org_)] were biosynthesized by a modified coprecipitation using two distinct routes: *in situ* process (ISP), in which functionalized γ‐Fe_2_O_3_ NPs formed directly in a solution rich in green tea (GT) extract at different weight/volume concentrations (x‐values), and after synthesis process (ASP), where preformed γ‐Fe_2_O_3_ NPs were subsequently functionalized with GT extract. The use of different salt‐precursors, precipitating agents, and reagent molarities resulted in NPs with distinct physical properties while maintaining good reproducibility across replicated samples, supporting the potential scalability of the biosynthesis process. The presence and relative quantity of polyphenols on the IONP surfaces were confirmed through ^57^Fe Mössbauer spectroscopy, TGA, and FTIR, which indicated an increase in polyphenol layer thickness with increase x‐values. Furthermore, the use of GT extract enabled a reduction of ≈5% in the amount of precipitation agents (NH_4_OH or NaOH) typically required in conventional coprecipitation, thereby minimizing post‐synthesis contaminant and environmental waste. Notably, increasing the polyphenol concentration in ISP—using FeCl_2_/FeSO_4_•7H_2_O and NH_4_OH or FeCl_2_/FeCl_3_ and NaOH—resulted in a decrease or increase in particle size, respectively, depending on the specific precursor combination. However, in all hybrid γ‐Fe_2_O_3_@GT(*t*_org_) samples, effective magnetic anisotropy values decreased with increasing x‐value. This trend is attributed to the reduction in dipole–dipole magnetic interactions, which are commonly observed in non‐functionalized NPs synthetized via coprecipitation. Functionalization was also evident in ASP samples, though ISP samples exhibited superior dispersion and greater suppression of interparticle magnetic interactions.

These findings demonstrate that variations in synthesis routes (ISP vs. ASP), salt precursors, and precipitating agents lead not only to differences in structural and morphological properties but also yield favorable magnetic characteristics, making the materials suitable for applications such as magnetic remediation of effluents, as already demonstrated in the literature.^[^ ^21^ ^]^ It is also worth noting that, even in organic‐rich environments, the Fe_3_O_4_ phase was observed only in few ISP samples shortly after synthesis. However, with aging and/or thermal annealing, these functionalized Fe_3_O_4_@GT(*t*_org_) NPs quickly transform into functionalized γ‐Fe_2_O_3_@GT(*t*_org_), which remained thermally stable up to 600 K. At 910 K, a partial transformation to α‐Fe_2_O_3_ (i.e., γ‐Fe_2_O_3_@α‐Fe_2_O_3_) was detected, and at 1173 K, the γ→α structural phase transition was completely. Therefore, the formation and functionalization of γ‐Fe_2_O_3_ NPs, using GT extract, proved to be a viable method for reducing chemical waste, enhancing the magnetic performance of Fe‐oxide NPs, enabling process scalability, and addressing several limitations reported in the literature. Consequently, this strategy can be considered a green synthesis route with promising potential for both industrial and environmental applications.

## Author Contributions

**Renzo Rueda‐Vellasmin**: conceptualization; methodology; software; validation; formal analysis; investigation; resources; data curation; writing—original draft preparation; writing—review and editing; visualization, **Juan A. Ramos‐Guivar**: conceptualization; methodology; software; validation; formal analysis; investigation; resources; data curation; writing—original draft preparation; writing—review and editing; visualization; supervision; project administration; **Jeferson Marques Santos**: methodology; validation; formal analysis; investigation; resources; data curation; writing—review and editing; visualization, **Noemi‐Raquel Checca‐Huaman**: software; validation; formal analysis; resources; data curation; writing—review and editing; visualization, **Edson C. Passamani**: conceptualization; methodology; software; validation; formal analysis; investigation; resources; data curation; writing—original draft preparation; writing—review and editing; visualization; supervision; project administration; funding acquisition. All authors have read and agreed to the published version of the manuscript.

## Conflict of Interest

The authors declare no conflict of interest

## Supporting information

Supplementary Material

## Data Availability

The data that support the findings of this study are available from the corresponding author upon reasonable request.

## References

[1] S. Malik , K. Muhammad , Y. Waheed , Molecules 2023, 28, 661.36677717

[2] N. Baig , I. Kammakakam , W. Falath , Mater. Adv. 2021, 2, 1821.

[3] A. Ali , H. Zafar , M. Zia , I. ul Haq , A. R. Phull , J. S. Ali , A. Hussain , Nanotechnol., Sci. Appl. 2016, 9, 49. 27578966 10.2147/NSA.S99986 PMC4998023

[4] Y. V. Alca‐Ramos , N.‐R. Checca‐Huaman , E. Arévalo‐Gardini , C. O. Arévalo‐Hernández , J. A. Ramos‐Guivar , Agriculture 2023, 13, 599.

[5] S. Arora , G. Murmu , K. Mukherjee , S. Saha , D. Maity , J. Biotechnol. 2022, 355, 21. 35752390 10.1016/j.jbiotec.2022.06.007

[6] O. V. Kharissova , H. V. R. Dias , B. I. Kharisov , B. O. Pérez , V. M. J. Pérez , Trends Biotechnol. 2013, 31, 240. 23434153 10.1016/j.tibtech.2013.01.003

[7] R. Hao , D. Li , J. Zhang , T. Jiao , Nanomaterials 2021, 11, 650. 33800123 10.3390/nano11030650 PMC8002084

[8] M. Coduri , P. Masala , L. Del Bianco , F. Spizzo , D. Ceresoli , C. Castellano , S. Cappelli , C. Oliva , S. Checchia , M. Allieta , D.‐V. Szabo , S. Schlabach , M. Hagelstein , C. Ferrero , M. Scavini , Nanomaterials 2020, 10, 867. 32365930 10.3390/nano10050867 PMC7279456

[9] T. Ahn , J. H. Kim , H.‐M. Yang , J. W. Lee , J.‐D. Kim , J. Phys. Chem. C 2012, 116, 6069.

[10] M. D. Carvalho , F. Henriques , L. P. Ferreira , M. Godinho , M. M. Cruz , J. Solid State Chem. 2013, 201, 144.

[11] L. Gloag , M. Mehdipour , D. Chen , R. D. Tilley , J. J. Gooding , Adv. Mater. 2019, 31,1904385. 10.1002/adma.201904385 31538371

[12] G. A. V. Magalhães‐Ghiotto , A. M. de Oliveira , J. P. S. Natal , R. Bergamasco , R. G. Gomes , Environ. Nanotechnol., Monit. Manag. 2021, 16, 100526.

[13] P. Mondal , A. Anweshan , M. K. Purkait , Chemosphere 2020, 259, 127509. 32645598 10.1016/j.chemosphere.2020.127509

[14] A. M. El Shafey , Green Proces. Synth. 2020, 9, 304.

[15] P. R. Chang , J. Yu , X. Ma , D. P. Anderson , Carbohydr. Polym. 2011, 83, 640.

[16] A. Bertelli , M. Biagi , M. Corsini , G. Baini , G. Cappellucci , E. Miraldi , Foods 2021, 10, 2595. 34828876 10.3390/foods10112595 PMC8621732

[17] V. C. Karade , P. P. Waifalkar , T. D. Dongle , S. C. Sahoo , P. Kollu , P. S. Patil , P. B. Patil , Mater. Res. Express. 2017, 4, 96102.

[18] J. Jeevanandam , Y. S. Chan , M. K. Danquah , ChemBioEng Rev. 2016, 3, 55.

[19] S. Dutta , S. K. Sivakamasundari , J. A. Moses , C. Anandharamakrishnan , in Nanoengineering in the Beverage Industry, Elsevier 2020, pp. 229–261.

[20] F. Zhu , S. Ma , T. Liu , X. Deng , J. Clean Prod. 2018, 174, 184.

[21] S. Saif , A. Tahir , Y. Chen , Nanomaterials 2016, 6, 209. 28335338 10.3390/nano6110209 PMC5245755

[22] P. Plachtová , Z. Medříková , R. Zbořil , J. Tuček , R. S. Varma , B. Maršálek , ACS Sustain Chem. Eng. 2018, 6, 8679. 30123724 10.1021/acssuschemeng.8b00986 PMC6093305

[23] J. A. R. Guivar , E. A. Sanches , F. Bruns , E. Sadrollahi , M. A. Morales , E. O. López , F. J. Litterst , Appl. Surf. Sci. 2016, 389, 721.

[24] G. Perez , M. P. Romero , E. B. Saitovitch , F. J. Litterst , J. F. D. F. Araujo , D. C. Bell , G. Solorzano , Solid State Sci. 2020, 106, 106295.

[25] C. Pecharromán , T. González‐Carreño , Juan E. Iglesias , Phys. Chem. Miner. 1995, 22, 21.

[26] H. Putz , K. Brandeburg , Match! ‐ Phase Identification from Powder Diffraction 2024.

[27] M. Y. Huertas‐Chambilla , M. F. Moyano‐Arocutipa , J. Y. Zarria‐Romero , N.‐R. Checca‐Huaman , E. C. Passamani , A. Arencibia , J. A. Ramos‐Guivar , Hyperfine Interact. 2022, 243, 24.

[28] Z. Klencsár , MossWinn 4.0i 2022.

[29] J. A. Ramos‐Guivar , D. A. Flores‐Cano , E. Caetano Passamani , Nanomaterials 2021, 11, 2310. 34578626 10.3390/nano11092310 PMC8471304

[30] A. Bandhu , S. Sutradhar , S. Mukherjee , J. M. Greneche , P. K. Chakrabarti , Mater. Res. Bull. 2015, 70, 145.

[31] J. A. Ramos‐Guivar , N.‐R. Checca‐Huaman , F. J. Litterst , E. C. Passamani , Nanomaterials 2023, 13, 1684. 37242100 10.3390/nano13101684 PMC10224039

[32] R. C. O’Handley , in Encyclopedia of Physical Science and Technology, Elsevier 2003, pp. 919–944.

[33] J. Xia , D. Wang , P. Liang , D. Zhang , X. Du , D. Ni , Z. Yu , Biophys. Chem. 2020, 256, 106282. 31756664 10.1016/j.bpc.2019.106282

[34] E. Giorgini , V. Notarstefano , R. Foligni , P. Carloni , E. Damiani , Foods 2023, 13, 109. 38201143 10.3390/foods13010109 PMC10778641

[35] C. Xiao , H. Li , Y. Zhao , X. Zhang , X. Wang , J. Environ. Manage. 2020, 275, 111262. 32858272 10.1016/j.jenvman.2020.111262

[36] K. K. Singh , K. K. Senapati , K. C. Sarma , J. Environ. Chem. Eng. 2017, 5, 2214.

[37] G. Socrates , Infrared and Raman Characteristic Group Frequencies: Tables and Charts, John Wiley & Sons, Chichester 2001.

[38] Y. Wu , S. Zeng , F. Wang , M. Megharaj , R. Naidu , Z. Chen , Sep. Purif. Technol. 2015, 154, 161.

[39] J. A. Ramos‐Guivar , E. O. López , J.‐M. Greneche , F. Jochen Litterst , E. C. Passamani , Appl. Surf. Sci. 2021, 538, 148021.

[40] R. M. Cornell , U. Schwertmann , The Iron Oxides: Structures, Properties, Reactions, Occurences and Uses, WILEY‐VCH GmbH & Co. KGaA, Weinheim 2003.

[41] S. Krepicka , K. Zaueta , Magnetic Oxides, Part 1, Wiley, Madison 1975.

[42] R. Rueda‐Vellasmin , N.‐R. Checca‐Huaman , E. C. Passamani , F. J. Litterst , J. A. Ramos‐Guivar , Hyperfine Interact. 2022, 243, 27.

[43] M. del P. Marcos‐Carrillo , N.‐R. Checca‐Huaman , E. C. Passamani , J. A. Ramos‐Guivar , Nanomaterials 2024, 14, 1607. 39404334 10.3390/nano14191607 PMC11478423

