# Supplementary material for: Tuning the Fe‐Oxide Nanoparticle Properties by Playing with Salt Precursors and Camellia sinensis Extract Concentrations
Source: Chemphyschem. 2025 Jul 23;26(18):e202500226. doi: 10.1002/cphc.202500226 (PMC12453295; doi:10.1002/cphc.202500226)
Supplement: Supplementary file 1 — Supplementary Material [file CPHC-26-e202500226-s001.pdf]

Tuning the Fe-oxide Nanoparticles Properties by Playing with Salt Precursors and *Camelia Sinensis* Extract Concentrations

Renzo Rueda-Vellasmin[1,*], Juan A. Ramos-Guivar[3], Jeferson Marques Santos[1], Noemi-Raquel Checca-Huaman[4], Edson C. Passamani[1,2,*]

[1]Programa de Pós-graduação em Física da Universidade Federal do Espírito Santo, Av. Fernando Ferrari, 514, Bairro Goiabeiras, Zip Code: 29075-910, Vitoria, ES, Brasil.

[2]Departamento de Física, Universidade Federal do Espírito Santo, Av. Fernando Ferrari, 514, Bairro Goiabeira, Zip Code: 29075-910, Vitoria, ES, Brasil

[3]Grupo de Investigación de Nanotecnología Aplicada para Biorremediación Ambiental, Energía, Biomedicina y Agricultura (NANOTECH), Facultad de Ciencias Físicas, Universidad Nacional Mayor de San Marcos, Av. Venezuela Cdra 34 S/N, Ciudad Universitaria, Lima 15081, Perú

[4]Centro Brasileiro de Pesquisas Físicas (CBPF), R. Xavier Sigaud, 150, Urca, Rio de Janeiro 22290-180, Brazil

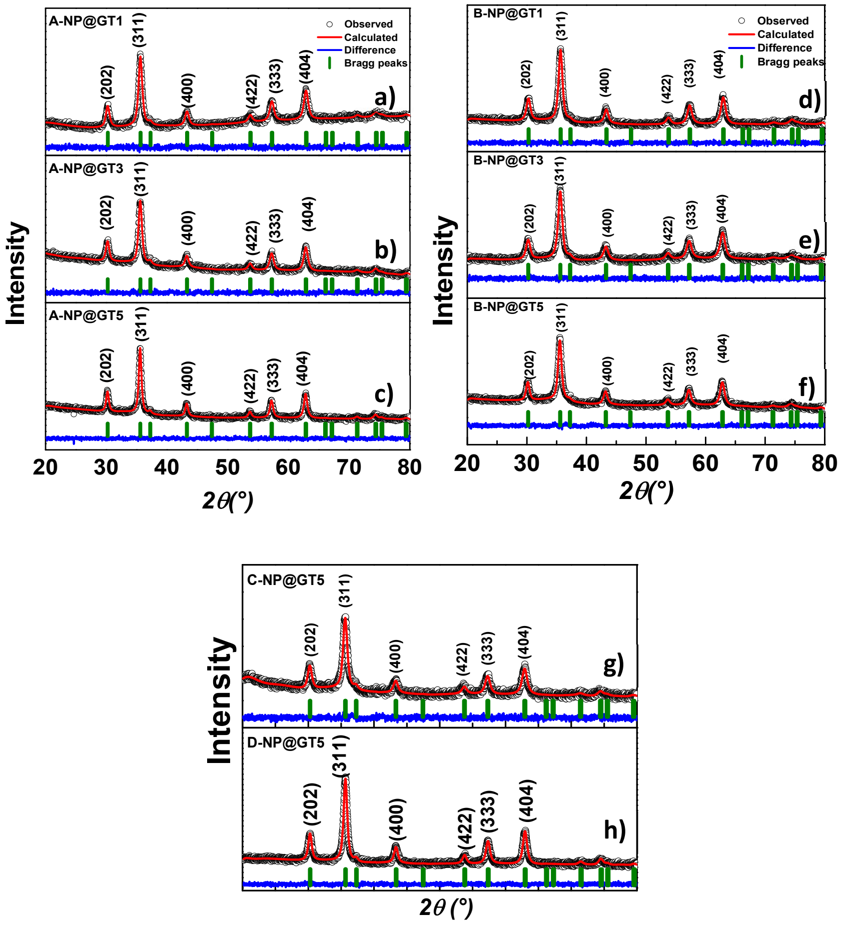

Figure S1 - Rietveld Refined XRD Diffractograms of some additional j-NP@GTx samples, with j- and x-values shown in the figure. The black marks are the XRD data of the material, red lines are the refined curves, green rods are the positions of the Miller planes or Bragg peaks and the blue lines are the difference between the refined and observed values.

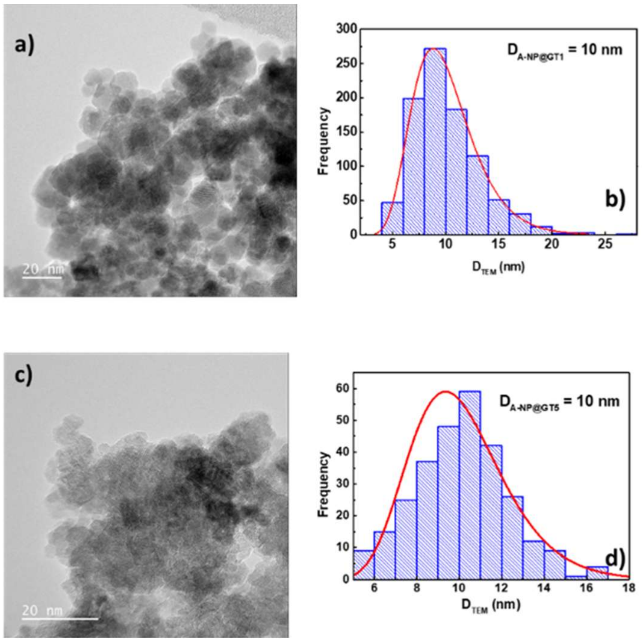

Figure S2 - TEM images (left-panel) and PSD histograms (right-panel) of the A-NP@GT1, a) and b), and A-NP@GT5 c) and d).

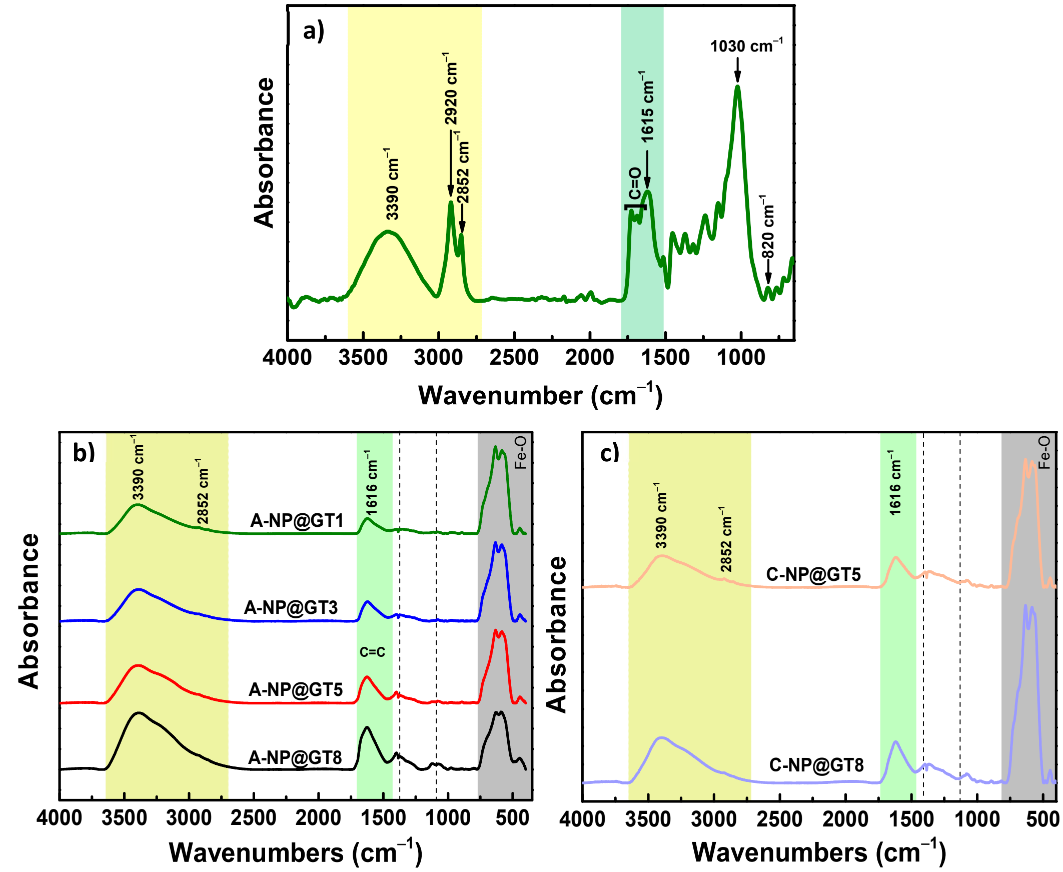

Figure S3 – a) FTIR spectrum of powdered green tea, where the yellow and green areas correspond to the vibrational modes of the organic compounds of the tea. In b) and c), the RT FTIR spectra for the A-NP@GTx, and C-NP@GTx samples, respectively.

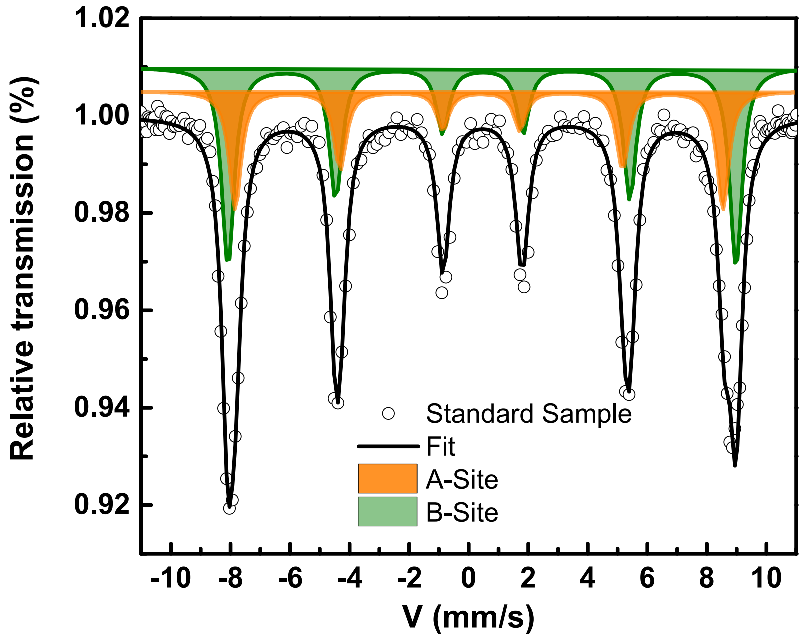

Figure S4 – 15 K Mössbauer spectrum of the bare $\gamma - Fe_2O_3$ NPs. Tetrahedral (A-site) and octahedral (B-site) subspectra of the spinel structure are also shown.

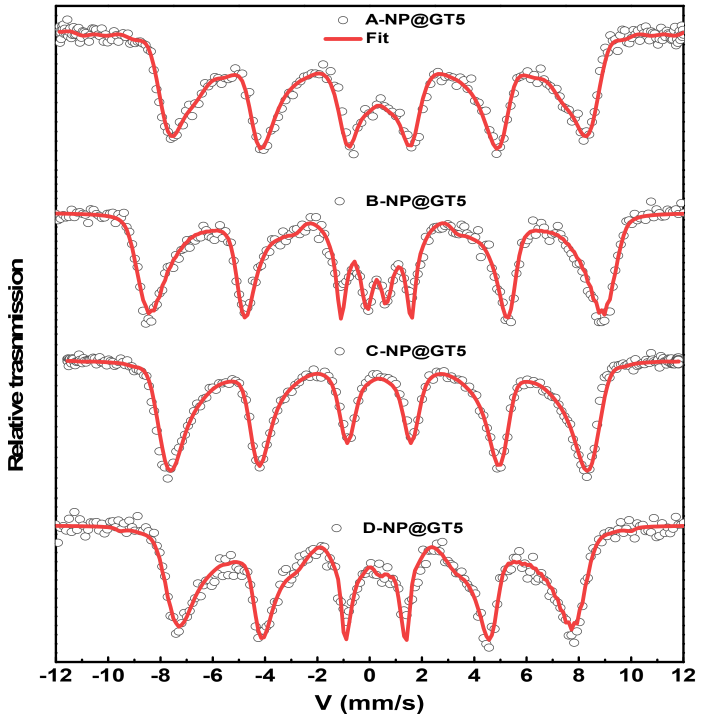

Figure S5 - RT Mössbauer spectra of the (a) A-NP@GT5, (b) B-NP@GT5, (c) C-NP@GT5, and (d) D-NP@GT5 samples. The experimental data (open circles) were fitted using a magnetic hyperfine field distribution (MHFD).

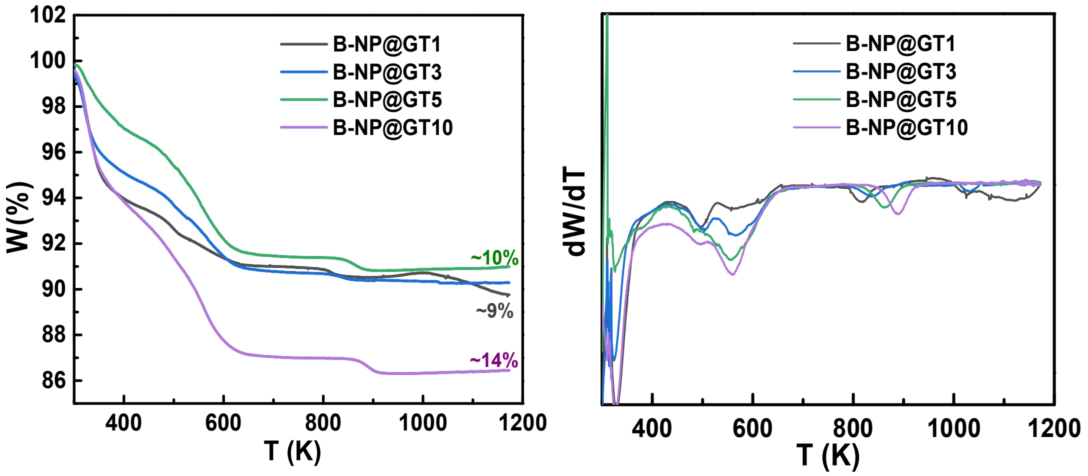

Figure S6 – (Left-hand side painle) TGA curves of the B-NP@GTx samples with x = 1, 3, 5 and 10. The right-hand side panel presents the corresponding derivative thermogravimetric curves (dW/dT), revealing better distinct thermal decomposition events primarily occurring between 300 K and 600 K.

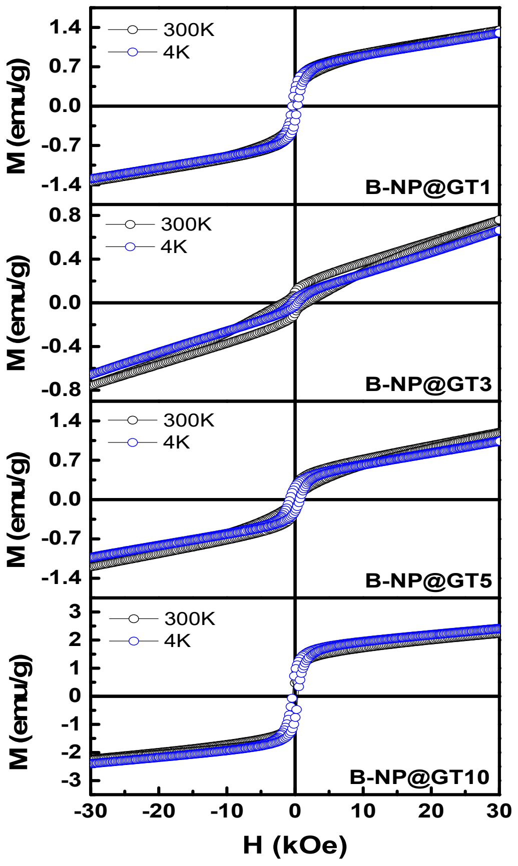

Figure S7 - 4 K *M*(*H*) loops of the B-NP@GT-x samples (x-values are indicated) annealed up to 900 K.

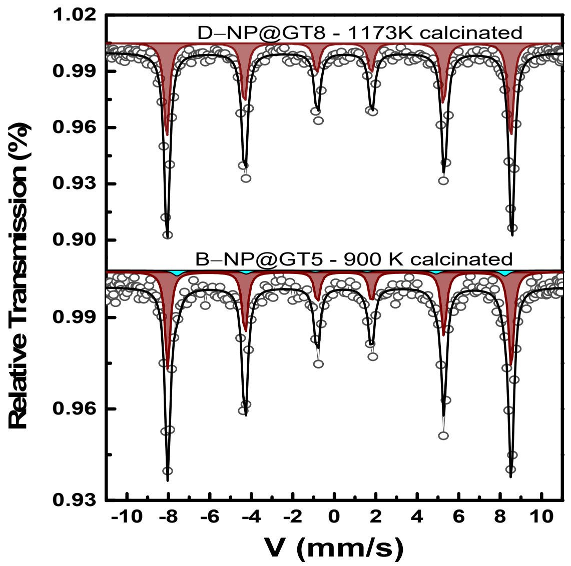

Figure S8 - RT Mössbauer spectra of the B-NP@GT5 after TGA analysis up to 900 K and up to 1173 K. The two components are the hematite (brown) and maghemite (cyan).

Table S1 - Structural parameters obtained from the refinements of the XRD diffractograms of the ISP (Series-A and B) and ASP (Series-C and D) samples (x-values are also shown).

Sample	Cell parameters (nm)	Average size	χ^2
	a=b=c;	(nm)	
	α=β=θ=90°	±1	
A-NP@GT1	0.8349 (1)	11	1.03
A-NP@GT3	0.8362 (1)	10	1.02
A-NP@GT5	0.8363 (1)	10	1.02
A-NP@GT8	0.8365 (1)	9	1.14
B-NP@GT1	0.8357 (1)	11	1.11
B-NP@GT3	0.8362 (1)	13	1.04
B-NP@GT5	0.8366 (1)	17	1.06
B-NP@GT10	0.8364 (1)	23	1.02
C-NP@GT5	0.8349 (1)	12	1.10
C-NP@GT8	0.8356 (1)	14	1.07
D-NP@GT5	0.8357 (1)	13	1.06
D-NP@GT10	0.8371 (1)	8	1.08

Table S2a - Hyperfine parameters extracted from the fittings of the Mossbauer spectra recorded at 300 K and 15 K of some samples of the A-NP@GTx and C-NP@GTx, where x-value are given in the table. ⟨IS⟩ is the mean isomer shift, ⟨2ϵ⟩ the quadrupolar shifting, and B_{hf} the peak magnetic hyperfine field distribution.

Samples	T (K)		$\langle IS \rangle$ (mm s^{-1}) ±0.02	$\langle 2\epsilon \rangle$ (mm s^{-1}) ±0.01	Γ (mm s^{-1}) ±0.01	B_{hf} (T) ±0.2	f (%) ± 1
Bare $\gamma - Fe_2O_3$ NPs	15	A	0.41	−0.05	0.49	50.8	36
		B	0.47	−0.02	0.50	52.9	64
**A-NP@GT1**	300	MHF D	0.36	----	0.30	41.0	100
	15	A	0.41	−0.01	0.50	50.8	44
		B	0.47	−0.02	0.56	53.1	49
		C	0.48	-0.01	0.56	48.7	5
**A-NP@GT3**	300	MHF D	0.36	---	0.30	41.0	100
	15	A	0.39	−0.01	0.46	50.9	32
		B	0.46	−0.04	0.54	52.8	61
		C	0.42	−0.01	0.56	48.9	7
**A-NP@GT5**	300	MHF D	0.36	----	0.30	41.0	100
	15	A	0.41	−0.01	0.49	51.1	34
		B	0.47	−0.02	0.56	52.9	54
		C	0.45	−0.02	0.58	48.5	12
**A-NP@GT8**	300*	MHF D	0.32	----	0.30	41.0	38
			0.32	0.74**	0.40	---	62
	15	A	0.41	−0.01	0.50	50.2	33
		B	0.46	−0.02	0.56	52.9	42
		C	0.47	−0.09	0.70	46.9	25
**C-NP@GT5**	300	MHF D	0.36	---	0.30	41.0	100
	15	A	0.49	−0.01	0.49	49.6	32
		B	0.46	−0.02	0.56	52.3	56
		C	0.49	−0.04	0.81	46.4	12
**C-NP@GT8**	300	MHF D	0.36	---	0.30	41.0	100
	15	A	0.42	−0.01	0.47	51.4	29

		B	0.46	−0.02	0.56	52.9	58
		C	0.39	−0.03	0.41	50.2	13

*This sample was fitted with two components (MHFD and doublet), ** indicates the quadrupolar splitting.

Table S2b - Hyperfine parameters extracted from the fittings of the Mossbauer spectra recorded at 300 K and 15 K of some samples of the B-NP@GTx and D-NP@GTx.

**Samples**	**T (K)**		**⟨*IS*⟩ (mm s^{-1}) ±0.02**	**⟨2ϵ⟩ (mm s^{-1}) ±0.01**	***Γ* (mm s^{-1}) ±0.01**	**B_{hf} (T) ±0.2**	***f* (%) ± 1**
**B-NP@GT1**	300	MHFD	0.36	---	0.30	41.0	100
	15	A	0.41	−0.05	0.51	50.8	34
		B	0.47	−0.02	0.56	52.9	59
		C	0.48	−0.03	0.56	47.9	7
**B-NP@GT3**	300	MHFD	0.36	---	0.30	41.0	100
	15	A	0.47	−0.01	0.42	53.3	35
		B	0.43	−0.05	0.59	51.4	56
		C	0.40	−0.01	0.56	48.2	9
**B-NP@GT5**	300	MHFD	0.36	---	0.30	41.0	100
	15	A	0.41	−0.01	0.51	51.2	30
		B	0.47	−0.02	0.56	52.8	59
		C	0.47	−0.01	0.52	48.2	11
**B-NP@GT10**	300	MHFD	0.32	----	0.30	41.0	100
	15	A	0.42	−0.01	0.51	51.4	25
		B	0.47	−0.02	0.56	52.8	53
		C	0.41	−0.03	0.50	49.8	22
**D-NP@GT5**	300	MHFD	0.36	---	0.30	41.0	100
	15	A	0.42	−0.01	0.51	51.4	32
		B	0.47	−0.02	0.56	52.8	56
		C	0.41	−0.03	0.56	49.8	11
**D-NP@GT10**	300	MHFD	0.36	---	0.30	41.0	100
	15	A	0.47	−0.01	0.49	51.1	31
		B	0.46	−0.02	0.54	53.1	60
		C	0.41	−0.01	0.52	49.6	9

Table S3 – Magnetic properties of the j-Series (j = A, B, C and D) samples obtained from the fittings of the 300 and 4 K M(H) curves with the LAS model mentioned in the text.

Sample	T (K)	*H_C* (kOe)	*M_S* (emu/g) ±1	*Mr* (emu/g)	*SQR Mr/Ms*	K_{eff} (10^4 J/m³)
A-NP@GT1	4	0.24	71	17	0.2	4.4
	300	-	62	---	---	3.4
A-NP@GT3	4	023	70	17	0.2	3.8
	300	-	60	---	---	2.9
A-NP@GT5	4	0.21	68	17	0.2	3.6
	300	-	56	---	---	2.9
A-NP@GT8	4	0.24	42	14	0.3	2.7
	300	-	32	---	---	2.1
B-NP@GT1	5	0.25	86	23	0.26	3.6
	300	-	75	---	---	2.8
B-NP@GT3	4	0.23	78	20	0.3	3.1
	300	-	68	---	---	2.3
B-NP@GT5	4	0.23	69	15	0.2	2.5
	300	-	59	---	---	1.9-2
B-NP@GT10	4	0.44	56	15	0.3	2.2
	300	0.04	49	6	0.1	1.7
C-NP@GT5	4	0.22	68	16	0.2	4.1
	300	-	61	---	---	3.3
C-NP@GT8	4	0.24	64	15	0.2	3.7
	300	-	57	---	---	3.0
D-NP@GT1	4	0.28	69	18	0.3	3.3
	300	-	61	---	---	2.7
D-NP@GT3	4	0.30	61	20	0.3	2.3
	300	-	58	---	---	2.2

D-NP@GT5	4	0.30	74	23	0.3	3.2
	300	-	66	---	---	2.7
D-NP@GT10	4	0.34	66	19	0.2	3.4
	300	-	62	---	---	3.0
